# Combination of flupentixol and melitracen tablets and rTMS improves post-stroke depression through neurovegetative symptoms changes: a retrospective analysis

**DOI:** 10.3389/fneur.2026.1773502

**Published:** 2026-05-19

**Authors:** Haizhou Wang, Kejun Zhang, Zikui Yang, Zhaokai Wang, Yanbo Cheng

**Affiliations:** 1 Department of Neurology, The Affiliated Hospital of Xuzhou Medical University, Xuzhou, Jiangsu, China; 2 First Clinical College of Xuzhou Medical University, Xuzhou, Jiangsu, China; 3 Department of Neurology, Xuzhou New Health Geriatric Hospital, Xuzhou, Jiangsu, China

**Keywords:** depression, flupentixol and melitracen tablets, neurovegetative symptoms, P300, post-stroke, repetitive transcranial magnetic stimulation

## Abstract

**Background:**

Post-stroke depression (PSD) is one of the most common neuropsychiatric complications among stroke survivors, with a substantial impact on functional recovery and quality of life. This study aimed to investigate the effect of flupentixol and melitracen tablets combined with repetitive transcranial magnetic stimulation (rTMS) on patients with post-stroke depression (PSD) and analyze the differences in the key factors of the 17-item Hamilton Depression Scale (HAMD_17_) scores.

**Methods:**

We conducted a retrospective analysis of 121 patients with PSD, including 57 patients who used the flupentixol and melitracen tablets alone and 64 patients who additionally received rTMS. General information was assessed. Follow-up indices after treatment included P300, National Institutes of Health Stroke Scale (NIHSS), HAMD_17_, and the Barthel Index (BI) and Pittsburgh Sleep Quality Index (PSQI) questionnaire. Observable mood (OM), cognitive symptoms (CS) and neurovegetative symptoms (NS), the three dimensions of HAMD_17_ was also analyzed. The concentrations of 5-hydroxytryptamine (5-HT), norepinephrine (NE), neuropeptide Y (NPY) and brain-derived neurotrophic factor (BDNF) in the serum were measured during all time points.

**Results:**

There was significant difference in the amplitude and latency of P300 and NS after 2 weeks and 4 weeks treatment between the groups, while there was significant difference in the concentrations of 5-HT, NE, NPY, BDNF and the scores of NIHSS, HAMD_17_, BI, PSQI after 4 weeks treatment. The treatment outcomes at 4 weeks demonstrated statistically significant differences compared to those observed at 2 weeks both at control group and combination group. The results also indicated that the treatment in the combined group demonstrated superiority over that in the control group with respect to both onset time and therapeutic efficacy.

**Conclusion:**

Combination of flupentixol and melitracen tablets and rTMS could significantly improve the depressive symptoms of patients with PSD, NS was the symptom dimension most closely related to the therapeutic response. More importantly, the P300 parameter might provide an early and objective indicator of the neurophysiological changes associated with the treatment.

## 1 Introduction

Stroke is a prevalent disorder of the central nervous system, ranking as the second leading cause of mortality and the third leading cause of disability globally (1). PSD represents the most prevalent neuropsychiatric comorbidity following ischemic stroke, affecting over one-third of stroke survivors. Individuals diagnosed with PSD frequently exhibit cognitive deficits, diminished quality of life, suicidal ideation, and a heightened risk of mortality (2). A meta-analysis of 61 studies encompassing 25,488 patients reported an overall incidence of depression at 31%, with cross-sectional prevalence rates of PSD estimated between 18 and 33% (3). The presence of PSD is associated with decreased patient engagement in rehabilitation training, which in turn affects the rehabilitation effect, and has become one of the urgent problems in the post-stroke rehabilitation process. Despite substantial evidence indicating that PSD is among the most severe complications following ischemic stroke, it remains frequently underrecognized and inadequately managed (4).

Currently, a variety of treatment approaches are available for PSD, with the primary modalities encompassing pharmacotherapy, social-psychological interventions and neuroregulatory techniques (5). Among the available treatment options for PSD, antidepressant medications are considered the preferred intervention, with flupentixol and melitracen tablets being one of the most commonly prescribed agents in clinical practice. However, their clinical utility is substantially limited by factors such as prolonged treatment duration, a high incidence of adverse effects, and suboptimal therapeutic outcomes (6). rTMS is a non-invasive neurostimulation technique that modulates the excitability of specific cerebral cortical regions by inducing localized electrical currents, thereby exerting therapeutic effects on depressive symptoms in stroke patients (7). However, the remission rate and therapeutic efficacy of rTMS in the treatment of PSD remain suboptimal, limiting its widespread clinical application (8). Studies have demonstrated that the combination of rTMS with flupentixol and melitracen tablets significantly improves therapeutic outcomes in patients with PSD compared to monotherapy with flupentixol and melitracen alone. Nevertheless, a subset of patients continues to exhibit persistent symptoms despite this combined intervention (9). Therefore, it is necessary to conduct a systematic evaluation of the clinical efficacy of rTMS in combination with flupentixol and melitracen tablets for the treatment of PSD. Assessing PSD solely based on the number or severity score of depressive symptoms may fail to account for the pathogenic heterogeneity across different symptom manifestations. Moreover, individuals with equivalent scale scores frequently display distinct depressive symptom dimensions (10). Therefore, the dimensional structure of depressive symptoms may serve as a potential predictor for treatment outcomes in patients with PSD.

P300 is a late-latency component of event-related potentials (ERP) and represents one of the most widely utilized biomarkers in clinical research. P300 primarily reflects neuroelectrophysiological alterations associated with cognitive and psychological processes, such as selective attention, working memory, and decision-making, during the brain’s engagement in cognitive tasks (11). It effectively excludes interference from external physical stimuli and subjective human factors, making it a widely adopted non-invasive technique for assessing cognitive processing in the brain (12).

This study aims to investigate the therapeutic efficacy of flupentixol and melitracen tablets combined with rTMS in patients with PSD, as well as to assess the predictive value of depressive symptom dimensions in determining treatment response. This study also investigates whether P300 demonstrates greater sensitivity than other rating scales, thereby serving as a more effective screening tool.

## 2 Method

### 2.1 Study participants

This retrospective study reviewed the medical records of patients with PSD who received treatment in the department of neurology at Xuzhou New Health Geriatric Hospital between January 2021 and December 2023. The inclusion criteria were: (1) age ≥ 18 years old; (2) met the diagnostic criteria for both stroke and depressive disorders by Diagnostic and Statistical Manual of Mental Disorders, Fifth Edition, Text Revision (DSM-5-TR); (3) the score of the HAMD_17_>8 points; (4) received the treatment of flupentixol and melitracen tablets or combined with rTMS. The exclusion criteria were: (1) diagnosing depression before stroke; (2) acute phase of stroke or traumatic brain injury, as well as pregnant women; (3) intracranial infection, intracranial effusion, or space-occupying lesions such as tumors; (4) epilepsy; (5) severe post-stroke complications, including pneumonia and cardiovascular diseases; (6) with a prior history of psychiatric disorders and substance use disorders; (7) the score of the Mini-Mental State Examination (MMSE)< 17 points or a score below level 3 on the Boston Diagnostic Aphasia Examination (BDAE). The control group consisted of patients diagnosed with post-stroke depression (PSD) who received standard stroke supportive care along with a once-daily oral dose of flupentixol/melitracen for 4 weeks. The combined group, in addition to the intervention of the control group, added rTMS, with 5 sessions per week for a total of 4 weeks. This report conforms to the World Medical Association Declaration of Helsinki and subsequent amendments. This study was approved by Xuzhou New Health Geriatric Hospital ethics committee (2025-03-018-K15), and informed consent was obtained from the patients.

### 2.2 Routine supportive treatments

Medications were administered to all participants in accordance with the latest clinical guidelines for stroke management, including neurotrophic therapy, improvement of cerebral circulation, antiplatelet agents, statins, antihypertensive drugs, and antidiabetic drugs (13). The antidepressant drug was flupentixol and melitracen tablets (0.5 mg/10 mg)/d, (H. Lundbeck A/S, Batch number: 2725154) one dose, for 4 weeks. Routine rehabilitation treatment was individually tailored by certified rehabilitation therapists according to each patient’s specific condition, including exercise training, occupational therapy, cognitive training, physical agent therapy, acupuncture, and psychological intervention, administered 5 days a week for a total duration of 4 weeks.

### 2.3 rTMS procedure

Stimulation was delivered through the VISHEE transcranial magnetic stimulator (Weisi Medical Technology Co., Ltd., Nanjing, Jiangsu, China) equipped with a figure-eight air-cooled coil (70 mm mean diameter). Patients were positioned in a semi-supine posture with the head immobilized throughout the stimulation procedure. Stimulation was delivered to the left dorsolateral prefrontal cortex. The active stimulation session was delivered at a frequency of 10 Hz and an intensity of 100% of the individual resting motor threshold (RMT), lasting 20 min and consisting of a total of 1,170 pulses (39 trains, each containing 30 pulses, with an inter-train interval of 28 s). Each patient received rTMS 5 days a week for a total duration of 4 weeks (except weekends).

### 2.4 Recording P300

ERP recording was conducted in a quiet, electromagnetically shielded room. Participants were seated in a comfortable chair in a relaxed state, wearing headphones, with their eyes closed, while remaining awake and attentive. The electroencephalographic signal was recorded at the parietal midline point electrode, which was positioned in accordance with the international 10 ~ 20 electrode placement system, with the linked A1 and A2 electrodes serving as Liu et al. (14). The ground electrode was positioned at the midpoint between the Fpz and Fz anatomical landmarks. The electrode impedance was maintained below 5 kΩ. The P300 component recorded at Pz was analyzed in terms of both amplitude and latency. The P300 amplitude was defined as the maximum positive voltage relative to a pre-stimulus baseline, measured within the time window of 250 to 500 ms following stimulus presentation. The P300 latency was quantified as the time interval between stimulus onset and the peak amplitude (15). The recording potential was averaged over 250 trials, with an analysis time window of 500 ms. High-pass and low-pass filters were set to 0.1 Hz and 30 Hz, respectively, and the sensitivity was calibrated at 10 μV/division.

### 2.5 Data collection and evaluations

General information of the two groups of patients was collected, including age, gender, BMI, type of lesion, site of onset, diabetes, hypertension, hyperlipidemia, coronary heart disease, history of stroke, smoking history, and drinking history. On the first, 14th, 28th day of hospitalization, a senior physician from the neurology department performed standardized assessments using the P300, NIHSS, HAMD_17_, and the BI. Sleep quality was evaluated using the PSQI questionnaire. The HAMD_17_ scale can be divided into three dimensions, namely OM, CS and NS (16). The therapeutic effect of PSD patients was evaluated based on the changes in HAMD_17_ scale scores before and after treatment. The efficacy of anti-PSD treatment was assessed based on the HAMD_17_ score reduction rate: a reduction of 75% or more is defined as complete recovery; a reduction of 50 to 74% is classified as significant recovery; a reduction of 25 to 49% is categorized as mild recovery; and a reduction of less than 25% is regarded as no effect. Blood samples were collected from patients in both groups in a fasting state prior to treatment initiation, as well as 2 and 4 weeks after the commencement of treatment. The collected blood samples were centrifuged at 3,000 g for 10 min to obtain serum. The concentrations of 5-HT and NE in the serum were measured using reversed-phase high-performance liquid chromatography. The concentrations of NPY and BDNF were measured using the enzyme-linked immunosorbent assay.

### 2.6 Statistical analysis

Statistical analysis of the data was performed using SPSS 25.0. Continuous variables were presented as mean ± standard deviation (SD). Normality tests (such as the Shapiro–Wilk test) and homogeneity of variance tests (such as the Levene test) were conducted for the continuous variables to verify the prerequisite assumptions for the *t*-test. Intergroup comparisons were conducted using independent samples *t*-tests. Paired samples *t*-tests were employed to compare data before and after treatment. Categorical variables were described as *n* (%) and analyzed using the chi-square (*χ*^2^) test or Fisher’s Exact test. In the main efficacy analysis, ANCOVA was used instead of the simple independent sample *t*-test for HAMD_17_ Scores 2 and 4 weeks after treatment.

## 3 Results

### 3.1 Demographic and clinical characteristics of patient in the two studied groups

According to the inclusion and exclusion criteria for the study, a total of 57 patients were assigned to the control group and 64 patients to the combination group from January 2021 to December 2023. There were no significant differences between the two groups with respect to age, gender, BMI, type of lesion, site of onset, diabetes, hypertension, hyperlipidemia, coronary heart disease, history of stroke, smoking history, and drinking history (all *p* > 0.05, Table 1). This balanced distribution of baseline demographics and clinical characteristics between the control and combination groups supports the comparability of subsequent efficacy analyse.

**Table 1:** Demographic and clinical characteristics.

	Control group	Combination group	*p*-value
Age (year)	58.3 ± 10.6	56.2 ± 8.7	0.234
Sex			
Male	37	46	0.408
Female	20	18	
BMI (kg/m^2^)	29.7 ± 5.4	31.2 ± 6.7	0.180
Type of lesion			
Hemorrhagic	16	25	0.201
Ischemic	41	39	
Site of onset			
Right	14	19	0.744
Left	21	24	
Both	22	21	
Diabetes	7	9	0.810
Hypertension	26	32	0.632
Hyperlipidemia	36	41	0.916
Coronary heart disease	7	10	0.600
History of stroke	6	8	0.799
Smoking history	27	38	0.188
Drinking history	34	42	0.500

BMI, body mass index.

### 3.2 Assessment of the efficacy degree in PSD patients after treatment

Assessment of the efficacy degree in PSD patients after treatment was shown in Table 2. The treatment outcomes at 4 weeks demonstrated statistically significant differences compared to those observed at 2 weeks both at control group and combination group. After a two-week treatment period, outcomes in the control group were as follows: 31 participants showed no effect, 13 exhibited mild recovery, 9 demonstrated significant recovery, and 4 achieved complete recovery. In the combination group, 12 participants showed no effect, 28 exhibited mild recovery, 16 demonstrated significant recovery, and 8 achieved complete recovery. After a four-week treatment period, outcomes in the control group were as follows: 8 participants showed no effect, 21 exhibited mild recovery, 13 demonstrated significant recovery, and 15 achieved complete recovery. In the combination group, 4 participants showed no effect, 7 exhibited mild recovery, 34 demonstrated significant recovery, and 19 achieved complete recovery. The results indicated that the treatment in the combined group demonstrated superiority over that in the control group with respect to both onset time and therapeutic efficacy. Notably, at the 4-week endpoint, the proportion of patients achieving ‘significant recovery’ or ‘complete recovery’ was substantially higher in the combination group (53/64, 82.8%) compared to the control group (28/57, 49.1%). Conversely, the rate of ‘no effect’ was markedly lower in the combination group (4/64, 6.3%) than in the control group (8/57, 14.0%).

**Table 2:** Assessment of the efficacy degree in PSD patients after treatment.

	Control group	Combination group	*p*-value
Two weeks after treatment			
Complete recovery	4	8	<0.001*
Significant recovery	9	16	
Mild recovery	13	28	
No effect	31	12	
Four weeks after treatment			
Complete recovery	15	19	<0.001*
Significant recovery	13	34	
Mild recovery	21	7	
No effect	8	4	
*p*-value	<0.001*	<0.001*	

<0.05*, significant difference; <0.01*, highly significant; <0.001*, extremely significant.

### 3.3 Efficacy assessments in patients with PSD

Efficacy assessments in patients with PSD was shown in Table 3. No statistically significant difference was observed in the scores of NIHSS, HAMD_17_, BI and PSQI between the two groups before treatment or at the 2-week follow-up post-treatment, while there was significant difference at the 4-week follow-up post-treatment. After adjusting for baseline NIHSS and BI, analysis of covariance revealed that the combined treatment group exhibited a significantly lower HAMD score compared with the single-drug treatment group at week 4 (*p*<0.01). A key finding was that electrophysiological changes preceded global clinical scales. Significant between-group differences in P300 latency, amplitude were already evident at the 2-week assessment (all *p* < 0.05), whereas differences in NIHSS, HAMD_17_, BI, and PSQI scores only reached significance at the 4-week assessment. This suggests that P300 parameters may serve as more sensitive early indicators of treatment response. At 4 weeks, the combination group showed significantly greater improvement than the control group across all assessed clinical, functional, sleep, and electrophysiological measures (all *p* < 0.05).

**Table 3:** Efficacy assessments in patients with PSD.

	Control group	Combination group	*p*-value
NIHSS			
First day of treatment	24.65 ± 6.48	25.15 ± 7.12	0.687
Two weeks after treatment	21.27 ± 5.61	20.57 ± 6.16	0.514
Four weeks after treatment	14.72 ± 4.32	11.28 ± 5.77	<0.001*
HAMD_17_			
First day of treatment	31.32 ± 5.33	29.79 ± 6.23	0.148
Two weeks after treatment	27.58 ± 6.28	25.77 ± 5.84	0.104
Four weeks after treatment	18.73 ± 6.28	14.99 ± 4.04	<0.001*
BI			
First day of treatment	61.44 ± 16.27	62.58 ± 15.63	0.696
Two weeks after treatment	67.28 ± 17.22	68.48 ± 16.62	0.698
Four weeks after treatment	72.25 ± 15.42	78.69 ± 16.67	0.029*
PSQI			
First day of treatment	17.17 ± 2.35	16.42 ± 3.42	0.159
Two weeks after treatment	15.42 ± 2.32	14.97 ± 2.03	0.261
Four weeks after treatment	12.43 ± 2.64	10.74 ± 2.77	<0.001*
P300 latency (ms)			
First day of treatment	357.48 ± 25.47	362.62 ± 21.35	0.235
Two weeks after treatment	336.62 ± 24.52	325.79 ± 26.64	0.021*
Four weeks after treatment	321.63 ± 25.68	311.27 ± 23.57	0.023*
P300 amplitude (μV)			
First day of treatment	3.96 ± 1.44	4.07 ± 1.36	0.667
Two weeks after treatment	4.46 ± 1.58	5.23 ± 1.66	0.010*
Four weeks after treatment	5.09 ± 1.47	5.87 ± 1.74	0.008*

NIHSS, National Institutes of Health Stroke Scale; HAMD_17_, 17-item the Hamilton Depression Scale; BI, the Barthel Index; PSQI, Pittsburgh Sleep Quality Index. <0.05*, significant difference; <0.01*, highly significant; <0.001*, extremely significant.

### 3.4 The relationship between the depressive symptom dimension and the efficacy in patients with PSD

The relationship between the depressive symptom dimension and the efficacy in patients with PSD was shown in Table 4. Of the three HAMD dimensions analyzed, only NS showed statistically significant between-group differences at both the 2-week and 4-week follow-ups (p* < 0.05), while no significant differences were found in OM and CS at any time point. This identifies NS as the principal dimensional factor associated with the superior treatment response observed in the combination group.

**Table 4:** The relationship between the depressive symptom dimension and the efficacy in patients with PSD.

	Control group	Combination group	*p-*value
OM			
First day of treatment	5.63 ± 1.27	5.72 ± 1.56	0.719
Two weeks after treatment	4.14 ± 1.35	4.57 ± 1.64	0.105
Four weeks after treatment	3.74 ± 1.36	3.87 ± 1.23	0.575
CS			
First day of treatment	17.57 ± 3.41	16.84 ± 3.17	0.225
Two weeks after treatment	15.43 ± 3.66	14.83 ± 4.26	0.393
Four weeks after treatment	7.29 ± 3.85	7.97 ± 4.68	0.350
NS			
First day of treatment	10.84 ± 3.94	10.25 ± 4.35	0.433
Two weeks after treatment	9.76 ± 2.33	6.34 ± 2.52	<0.001*
Four weeks after treatment	6.37 ± 2.14	4.97 ± 1.68	<0.001*

OM, observable mood; CS, cognitive symptoms; NS, neurovegetative symptoms. <0.05*, significant difference; <0.01*, highly significant; <0.001*, extremely significant.

### 3.5 Comparison of neurotransmitter and BDNF in the two groups

The comparative analysis of 5-HT, NE, NPY and BDNF levels across different time points was presented in Table 5. Levels of all four biomarkers increased progressively over time within each group. More importantly, at the 4-week assessment, the combination group exhibited significantly greater increases in the concentrations of 5-HT, NE, NPY, and BDNF compared to the control group (all *p* < 0.001), suggesting that the combined therapy had a more potent effect on modulating neurochemical and neurotrophic pathways.

**Table 5:** Comparison of neurotransmitter and BDNF in the two groups.

	Control group	Combination group	*p*-value
5-HT (ng/L)			
First day of treatment	109.42 ± 16.20	105.43 ± 18.85	0.212
Two weeks after treatment	116.94 ± 13.66	120.87 ± 16.28	0.151
Four weeks after treatment	126.54 ± 19.74	146.46 ± 15.64	<0.001*
NE (ng/L)			
First day of treatment	28.47 ± 3.62	29.02 ± 4.19	0.440
Two weeks after treatment	37.84 ± 6.42	39.56 ± 5.22	0.111
Four weeks after treatment	47.65 ± 5.41	59.77 ± 8.23	<0.001*
NPY (ng/L)			
First day of treatment	114.66 ± 15.28	119.33 ± 12.86	0.074
Two weeks after treatment	138.86 ± 16.43	142.69 ± 17.92	0.222
Four weeks after treatment	154.61 ± 15.69	175.81 ± 16.05	<0.001*
BDNF (μg/L)			
First day of treatment	7.19 ± 0.74	6.97 ± 0.94	0.153
Two weeks after treatment	9.07 ± 1.74	9.46 ± 1.83	0.231
Four weeks after treatment	11.71 ± 1.32	14.64 ± 1.44	<0.001*

5-HT, 5-hydroxytryptamine; NE, norepinephrine; NPY, neuropeptide Y; BDNF, brain-derived neurotrophic factor. <0.05*, significant difference; <0.01*, highly significant; <0.001*, extremely significant.

## 4 Discussion

PSD was defined as a syndrome that develops following a stroke, characterized by a range of depressive symptoms accompanied by associated somatic manifestations. As one of the most prevalent and manageable post-stroke complications, PSD required timely identification and intervention. Failure to diagnose and treat it promptly might impair the patient’s neurological recovery and hinder their reintegration into society (17). The pathogenesis of PSD was highly complex and has not yet been fully elucidated. PSD could manifest during the acute phase (<1 month), intermediate phase (1 ~ 6 months), and recovery phase (>6 months) following a stroke, the incidence rates were 33, 33 and 34%, respectively (18). Numerous studies had demonstrated that PSD was significantly associated with adverse outcomes following stroke. It not only contributed to prolonged hospitalization, impaired neurological recovery, and a greater decline in independence in daily living, but also linked to increased mortality rates.

Flupentixol and melitracen tablets were a compound formulation containing the active components flupentixol and melitracen, both of which exhibited potent antidepressant properties. This medication had been extensively utilized and supported by clinical evidence in recent years (19). rTMS was a novel, painless, non-invasive therapeutic technique. It operated by delivering repeated magnetic pulses to a targeted cortical region of the brain, thereby modulating physiological functions. When administered properly in accordance with established protocols, this intervention was considered safe and was not associated with significant adverse effects (20). To evaluate the efficacy of the combined treatment, we conducted a comparative analysis, which demonstrated that the combined treatment exhibited significantly greater effectiveness compared to pharmacotherapy alone. What’s more, to explore the clinical characteristics and associated predictive factors in this patient population hold significant value for informing clinical decision-making in diagnosis and treatment.

To identify predictive factors associated with the therapeutic efficacy of rTMS combined with flupentixol and melitracen tablets in patients with PSD, this study analyzed the three dimensions of the HAMD_17_. The results indicate that NS served as a significant predictive factor. The NS dimension, which belonging to physiological and vegetative functions, might reflect the severity of the depressive state in patients with PSD. Several studies have demonstrated that rTMS can promote the proliferation, differentiation, and migration of neural stem cells NSCs, while also inhibiting their apoptosis (21). The restoration of impaired neural function led to an improvement in neurotransmitter levels at neuronal synapses. This also explains the basis for our determination of the concentrations of NPY and BDNF.

5-HT, NE, and NPY were neurotransmitters that are widely distributed in the central nervous system and were closely implicated in the onset and progression of depression. BDNF was a neurotrophic protein that played a crucial role in maintaining neural homeostasis. In this study, the combined group exhibited significantly greater increases in the levels of 5-HT, NE, NPY, and BDNF compared to the control group at 4 weeks after treatment, indicating that the combination of flupentixol and melitracen tablets and rTMS had a more pronounced effect on modulating neurotransmitter secretion and enhancing neurological function in patients with PSD. Potential explanations for this enhanced efficacy might lie in the complementary neuronal regulatory mechanisms through which drugs and rTMS exert their antidepressant effects.

The P300 component was the most prominent endogenous late potential within event-related potentials. It was minimally affected by external physical stimuli or extraneous human factors, yet demonstrated high sensitivity to psychological variables. As such, it was widely regarded as a reliable biomarker for assessing cognitive functioning and mental processes (22). Our findings that P300 latency and amplitude demonstrated significant between-group differences as early as 2 weeks post-treatment, preceding the separation in traditional clinical scales (HAMD, NIHSS, BI, PSQI) by 2 weeks, underscore its potential as a sensitive and early objective biomarker for monitoring therapeutic response in PSD. This temporal advantage is of paramount clinical significance. Early detection of treatment response can guide timely therapeutic adjustments, potentially improving outcomes and preventing prolonged ineffective interventions. The objective nature of P300, being less susceptible to rater bias or patient subjectivity than symptom scales, addresses a critical limitation in PSD assessment, especially in patients with aphasia or cognitive impairment who may have difficulty completing self-reports (23). This observation also offered a plausible explanation for the significant differences observed in HAMD_17_ scores and P300 at the two-week treatment interval in our study. Meanwhile, this finding provided strong evidence supporting the validity of P300 as a sensitive and reliable indicator.

The utility of P300 extends beyond mere early detection. A growing body of evidence positions P300 as a robust electrophysiological correlate of cognitive dysfunction in PSD. Meta-analyses have consistently shown that compared to both healthy controls and stroke patients without depression, individuals with PSD exhibit significantly prolonged P300 latency and reduced amplitude. These alterations are thought to reflect impairments in attention allocation, context updating, and working memory—core cognitive domains frequently affected in PSD. Therefore, the improvement in P300 parameters observed in our combination group likely signifies not only a reduction in depressive symptomatology but also a concurrent amelioration of underlying cognitive deficits, which are crucial for functional recovery and quality of life (24, 25).

The pathophysiological basis for P300 changes in PSD and their modulation by treatment may be linked to the neurobiological alterations targeted by our intervention. P300 generation involves a distributed network including the prefrontal cortex, temporal–parietal junction, and anterior cingulate cortex—regions implicated in both mood regulation and cognitive control. Dysfunction in these networks, potentially due to monoaminergic dysregulation and reduced neuroplasticity, is a hallmark of depression. The concordant improvement in P300 and neurochemical markers supports the hypothesis that the combined therapy facilitates the restoration of neural network efficiency and synaptic integrity, which is electrophysiologically captured by P300 normalization (26).

The findings of this study offer multiple implications for the clinical management of PSD. Firstly, the application potential of P300 as an early and objective biomarker for therapeutic efficacy is significant. Traditional scale assessments are subjective and usually take several weeks to show changes, while P300 parameters (latency and amplitude) can detect differences between groups as early as 2 weeks after treatment. This provides a possible “decision support tool” for clinicians: if the P300 indicators do not show improvement in patients within 2 weeks after combined treatment, it may suggest that an early re-evaluation or adjustment of the treatment plan is necessary to avoid long-term exposure to potentially ineffective interventions and achieve more dynamic and individualized treatment management. This is particularly valuable for PSD patients with aphasia and cognitive impairments who cannot complete subjective scale assessments. Secondly, the neurovegetative symptom dimension has been identified as a key predictor of therapeutic response. This suggests that clinical assessment should go beyond the total score of depression and pay more attention to changes in the NS dimension. In clinical practice, targeted assessment and monitoring of NS symptoms at the beginning of treatment may help predict the long-term rehabilitation trajectory of patients earlier. Moreover, the synergistic effect of the combined treatment regimen (rTMS combined with fluoxetine and mirtazapine) in improving NS, P300, and neurobiochemical indicators has provided a more evidence-based and strengthened treatment strategy for PSD patients who do not respond well to single drug treatment.

However, this study had several limitations. First, this is a retrospective, single-center study with a limited sample size, which inherently increases the risk of selection bias and residual confounding. Second, treatment delivery varied across participants, even with protocol adherence, due to inconsistent rTMS application and pharmacotherapy dosing, and no individual-level monitoring was conducted. This variability may bias efficacy estimates. Third, the absence of rTMS-only or sham rTMS standard careprecluded definitive conclusions regarding the treatment-specificity of these predictive effects. Fourth, the 4-week follow-up is too short to assess long-term treatment efficacy or late-emerging adverse effects. What’s more, using the rate of HAMD_17_ score reduction as an indicator to evaluate the therapeutic effect of anti-PSD treatment has not yet gained widespread recognition from the international academic community, and we cannot completely distinguish neurovegetative symptoms from stroke-related somatic symptoms. This study exploratorily assessed the efficacy of combined treatment without multiple comparison correction (such as Bonferroni). Thus, findings significant at *p* < 0.05, are preliminary and require replication in independent samples. Future prospective randomized controlled trials with longer follow-up, active comparators, and finer symptom assessment are needed to confirm these results and clarify underlying mechanisms.

## 5 Conclusion

In summary, combination of flupentixol and melitracen tablets and rTMS could significantly improve the depressive symptoms of patients with PSD, NS was the symptom dimension most closely related to the therapeutic response. More importantly, the P300 parameter might provide an early and objective indicator of the neurophysiological changes associated with the treatment.

## Data Availability

All data generated or analyzed during this study are included in this published article. Requests to access these datasets should be directed to wanghaoyu20220202@163.com.
